# Assessment of folate receptor alpha and beta expression in selection of lung and pancreatic cancer patients for receptor targeted therapies

**DOI:** 10.18632/oncotarget.23321

**Published:** 2017-12-15

**Authors:** Jiayin Shen, Yingwen Hu, Karson S. Putt, Sunil Singhal, Haiyong Han, Daniel W. Visscher, Linda M. Murphy, Philip S. Low

**Affiliations:** ^1^ Department of Chemistry, Purdue University, West Lafayette, IN, USA; ^2^ Institute for Drug Discovery, Purdue University, West Lafayette, IN, USA; ^3^ Division of Thoracic Surgery, Department of Surgery, University of Pennsylvania School of Medicine, Philadelphia, PA USA; ^4^ Clinical Translational Research Division, The Translational Genomics Research Institute, Phoenix, AZ, USA; ^5^ Department of Laboratory Medicine and Pathology, Mayo Clinic College of Medicine, Rochester, MN, USA; ^6^ Department of Biochemistry and Molecular Biology, Mayo Clinic College of Medicine, Rochester, MN, USA

**Keywords:** folate receptor alpha, folate receptor beta, lung cancer, pancreatic cancer, tumor associated macrophages

## Abstract

A number of folate receptor (FR) targeted small molecular drugs and monoclonal antibodies have been introduced into clinical trials to treat FR positive cancers. Because the therapeutic efficacy of these drugs depends prominently on the level of FR-α expression on the cancer cells, patients have been commonly selected for FR-targeted therapies based on the intensity of a folate-targeted radioimaging agent. Unfortunately, uptake of such imaging agents can be mediated by both major isoforms of the folate receptor, FR-α and FR-β. Logically, if the FR positive cell population in a tumor mass is dominated by FR-β positive macrophages, patients could be selected for therapy that have few FR-expressing cancer cells. Although several IHC studies have examined expression of either FR-α or FR-β, no study to date has investigated expression of both FR-α and FR-β in the same tumor mass. Herein, we utilize monoclonal antibodies specific for FR-α (mAb343) and FR-β (m909) to query each isoform's expression in a range of cancers. We show that lung and pancreatic adenocarcinomas express the full spectrum of FR-α and FR-β combinations with ~76% of lung adenocarcinomas expressing both FR-α and FR-β while pancreatic cancers express primarily FR-β. Thus, while folate-targeted imaging of lung cancer patients might accurately reflect the expression of FR-α on lung cancer cells, imaging of pancreatic cancer patients could mislead a physician into treating a nonresponding patient. Overall, these data suggest that an independent analysis of both FR-α and FR-β should be obtained to predict the potential efficacy of a folate-targeted drug.

## INTRODUCTION

Folate receptors (FR) are one family of membrane-anchored receptors that participate in the transport of folate into cells [1]. Four isoforms of FR (α, β, γ and δ) have been identified, with each displaying a unique expression pattern in normal tissues. FR-α is primarily found on the apical surfaces of epithelial cells in the mammary ducts, lungs, kidneys and the choroid plexus [2–4], whereas FR-β has only been found on activated but not resting or quiescent myeloid cells [5, 6]. FR-γ is a predominately secreted receptor that is found in very low concentrations in the blood [7, 8], while FR-δ is only known to be expressed on ova and regulatory T-cells [9].

Unlike this restricted FR expression in normal tissues, a broad range of cancers express one or more FRs. FR-α has been found on cancers of the ovary, breast, head & neck, endometrium, lung, bladder, pancreas, colon and kidney [10–21], with an estimated 40% of human cancers overexpressing the receptor. More recently, FR-β has been shown to be expressed on both malignant cells and tumor-associated macrophages from a wide variety of cancers, including cancers of the lung, breast, liver, brain, uterus, thyroid, stomach, ovary, head & neck, skin, kidney, pancreas, bladder, cervix, esophagus, prostate, testis, and colon [6, 22]. In fact, over 25% of the tumor sections examined exhibited FR-β expression on the cancer cells and over 50% had expression on their tumor-associated macrophages [22].

Due to upregulation of FRs in many tumors, multiple diagnostic and therapeutic agents have been targeted to an FR for imaging or treating the FR positive cancer [23–30]. In fact, six folate-targeted drugs [26–30], four folate-targeted imaging agents [31, 32] and an FR-α specific monoclonal antibody [30, 33] have been introduced into human clinical trials over the last several years. In many of these trials, patients have been selected for an FR-targeted therapy based on the intensity of their tumor's uptake of a folate-targeted radioimaging agent. Unfortunately, these images do not distinguish whether imaging agent retention is mediated by FR-α or FR-β, and hence a patient could be selected for folate-targeted chemotherapy when most of the imaging agent uptake was by FR-β positive macrophages. Because FR-β positive macrophages do not respond to most antimitotic drugs and since elimination of such macrophages would also not directly eradicate the cancer cells, it is conceivable that some patients will have been inaccurately selected for an FR-targeted chemotherapy because their tumors were replete with macrophages.

Unfortunately, there is currently no published information on the independent expression of FR-α and FR-β in the same tumor sample. In fact, the only published data relevant to this topic reports the binding of an antibody with reactivity towards both FR-α and FR-β in ovary and lung carcinomas [34] and the FR-α and FR-β mRNA levels in ovarian and fallopian adenocarcinomas [11], which often correlate poorly with protein expression [35, 36], leaving considerable uncertainty over the relative FR protein levels in these cancers. In an attempt to obtain an initial indication regarding the levels of FR-α and FR-β proteins in the same tumor samples, we employed two monoclonal antibodies with non-overlapping specificities for FR-α and FR-β. Quantitative analyses of IHC stains of a large number of lung and pancreatic cancer specimens reveal that both FR-α and FR-β are commonly upregulated in both malignancies and that in many cases, the level of FR-β expression exceed that of FR-α. These data argue for the need to develop separate imaging agents for both FR-α and FR-β.

## RESULTS

### Antibodies

**Figure 1:**
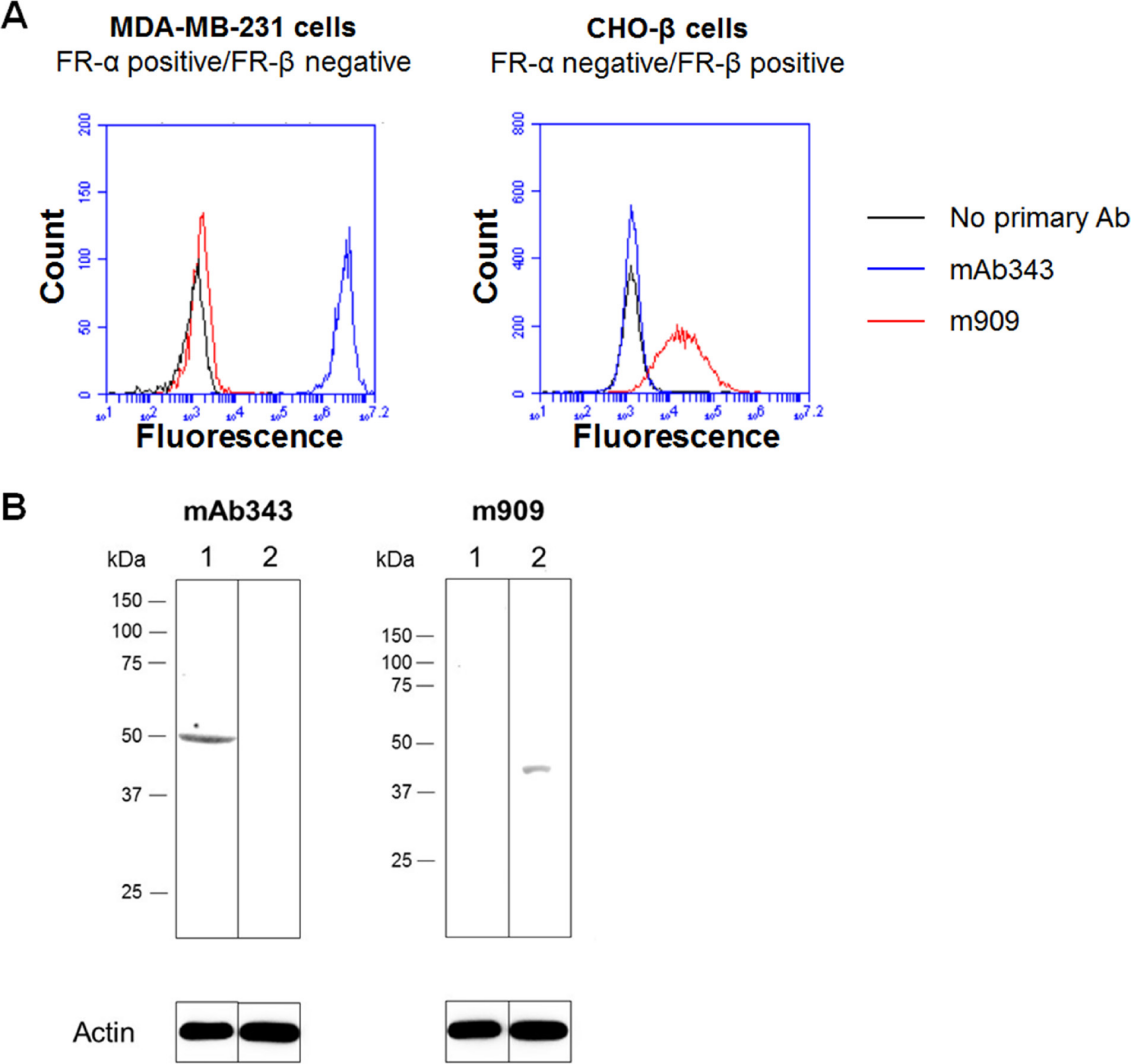
FR isoform specificity of antibody staining (**A**) MDA-MB-231 (FR-α positive/FR-β negative) and transfected CHO- β cells (FR-α negative/FR-β positive) were incubated with mAb343 (blue line), m909 (red line) or no primary antibody (black line) for 30 minutes at room temperature. Cells were washed and incubated with secondary antibody for 30 min at room temperature. Cells were washed again and analyzed by flow cytometry. (**B**) Lysate from FR-α positive/FR-β negative cell line MDA-MB-231 (lane 1) and the FR-α negative/FR-β positive transfected cell line CHO-β (lane 2) were ran on a gel, transferred to nitrocellulose, blocked overnight at 4^°^C, washed, and blotted with mAb343 or m909 overnight at 4^°^C. After washing again, blots were incubated with HRP-conjugated secondary antibody for 1 hour at room temperature. A luminescent HRP substrate was added and blots were visualized.

The monoclonal antibodies for use in staining tissue sections for FR-α (mAb343) and FR-β (m909) were selected based on their abilities to specifically stain their respective FR isoforms in a previous publication [22, 37]. However, in order to confirm their FR isoform specificities, cell lines that express only FR-α (MDA-MB-231) or only FR-β (transfected CHO-β) were used to query binding via both flow cytometry and western blotting. As shown in Figure 1, mAb343 only bound to MDA-MB-231 cells, while m909 only bound to CHO-β cells. These results agree with prior studies and confirm that binding of mAb343 and m909 is specific for FR-α and FR-β, respectively.

### Multi-cancer TMA

In order to investigate the expression of FR-α and FR-β in the same tumor specimens, sequential tissue sections were stained with mAb343 and m909 as outlined in the Methods. The staining intensity of each monoclonal antibody was then assessed by a trained pathologist and scored on a scale of 0 to 3, where 0 indicated no visible staining and 3 designated the highest level of staining.

**Figure 2:**
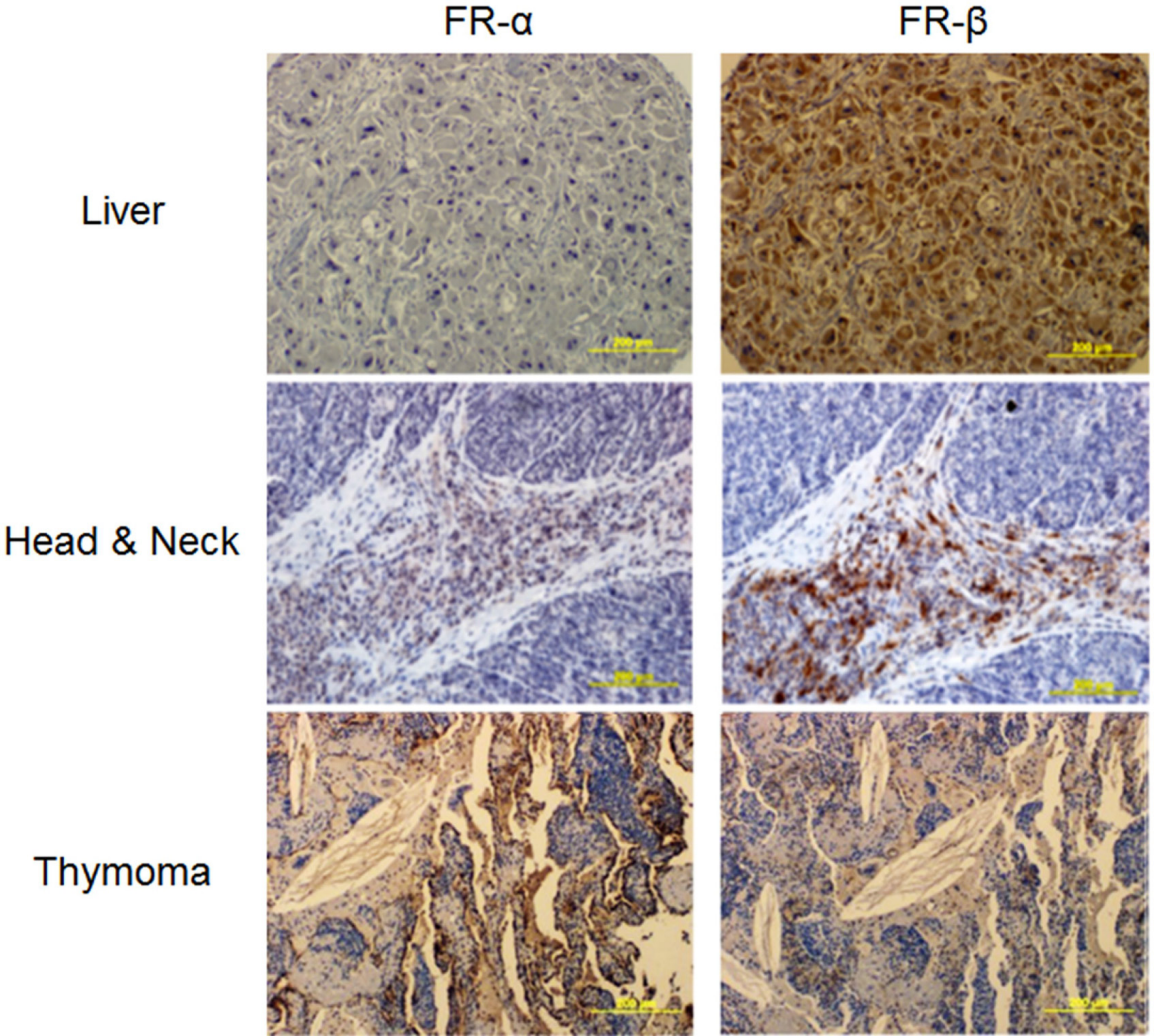
Representative images of various neoplastic tissues sequentially stained with the FR-α and FR-β specific monoclonal antibodies mAb343 and m909, respectively

Initially, IHC staining was performed on a broad multi-cancer tumor microarray (TMA) containing ~8–10 sections per cancer type and 8 total cancer types. FR-α and/or FR-β staining was observed in most specimens (see representative stains in Figure 2), suggesting that FR-targeted imaging agents should reveal the locations and sizes of most of these human cancers. Unfortunately, variability in FR-α and FR-β staining was so large in these samples that statistically significant conclusions could not be drawn from the limited number of samples available. Therefore, much larger arrays of lung (212 different tumors) and pancreatic (64 different tumors) cancer TMAs that contained adjacent tissue sections were obtained and used for statistical evaluation of FR-α and FR-β expression.

### Lung adenocarcinoma TMA

**Figure 3:**
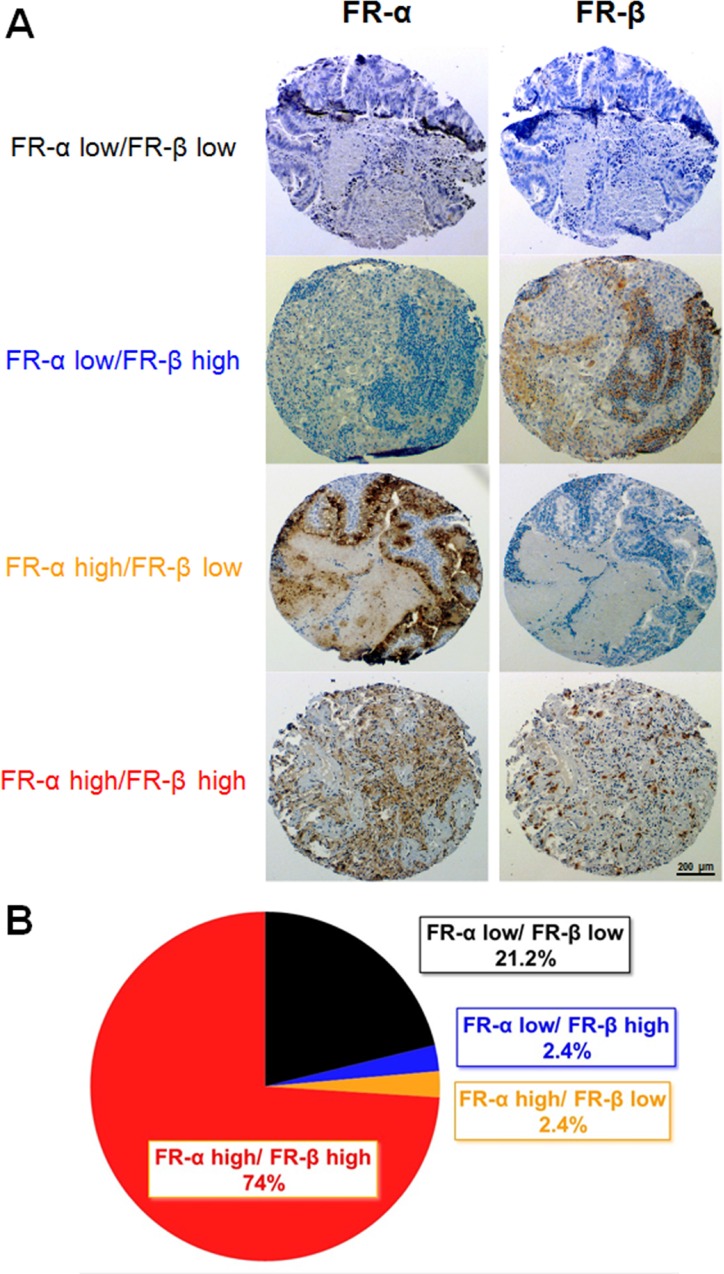
FR-α and FR-β expression in lung cancer (adenocarcinomas) tissue sections Sequential sections were stained with mAb343 or m909. (**A**) Representative images of each level of FR-α and FR-β staining is shown. (**B**) Summary of the percentage of tissue sections staining positive for FR-α and FR-β (*n* = 212).

As shown in Figure 3, expression of FR-α and FR-β in lung adenocarcinoma sections varied significantly, resulting in all possible ratios of FR-α to FR-β expression (i.e., FR-α low/FR-β low, FR-α low/FR-β high, FR-α high/FR-β low, FR-α high/FR-β high). When analyzed individually, 76% of the 212 total tumor sections (i.e. 162 samples) stained positive for FR-α and 76% also stained positive for FR-β. Importantly, this percentage of FR-α and FR-β positive tumors is similar to that reported in previous studies that simply examined each receptor individually [16, 22, 37]. When both FR-α and FR-β were subsequently analyzed on the same tumor specimen, however, the vast majority (97%) of tumors that expressed one FR isoform also expressed the other. Similarly, nearly every tumor that expressed few or no FR-α also expressed few or no FR-β. Closer inspection of the FR positive tumors further revealed that FR-α and FR-β rarely colocalized to the same region of the malignant lesion, with FR-α usually distributed throughout the cancer mass and FR-β commonly clustered in regions of high immune cell infiltration. Not surprisingly, the majority of FR-α appeared to reside on cancer cells while most of FR-β was localized to macrophage-like cells.

### Staining of pancreatic TMAs

**Figure 4:**
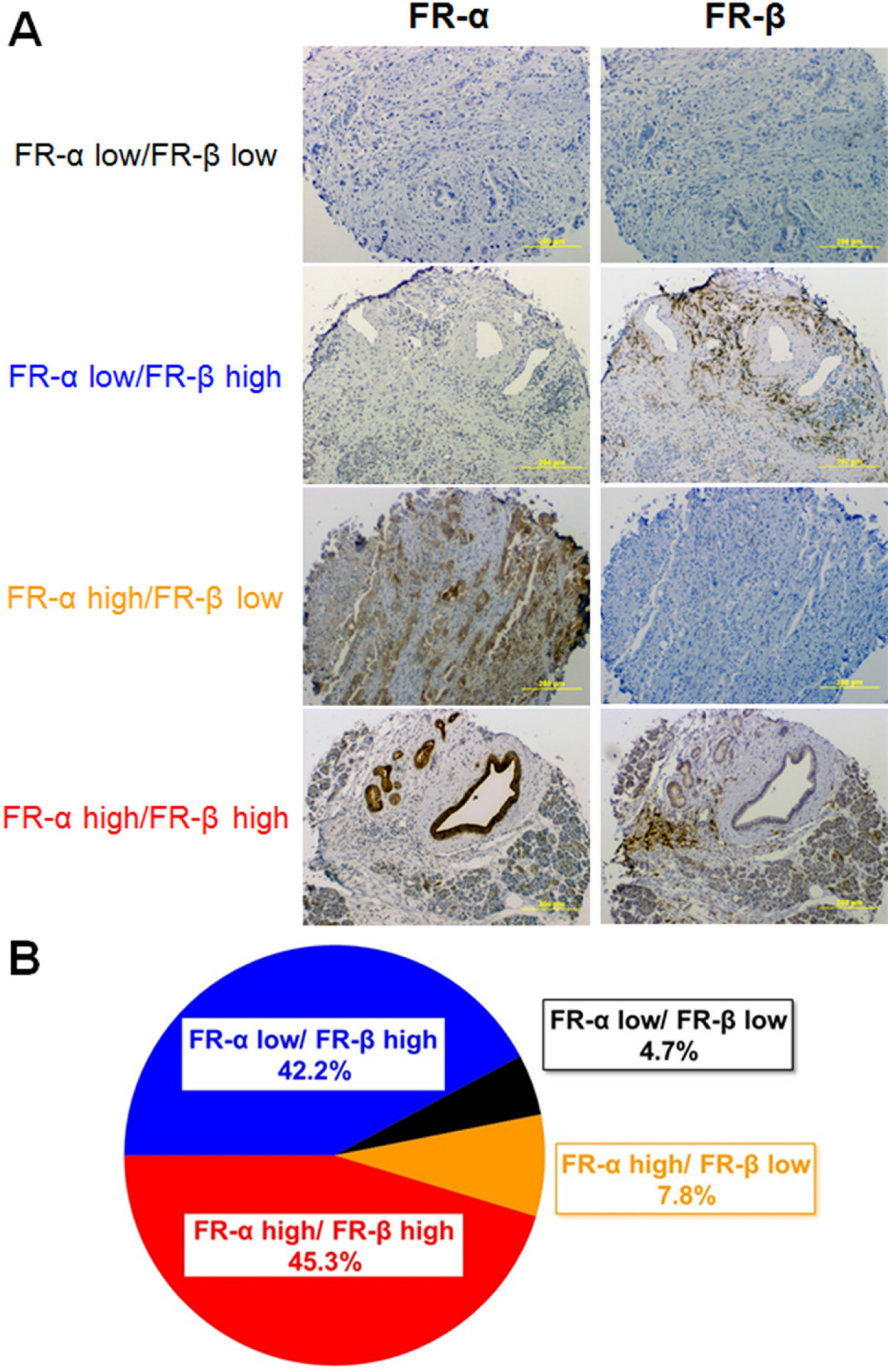
FR-α and FR-β expression in pancreatic cancer tissue sections Sequential sections were stained with mAb343 or m909. (**A**) Representative images of each level of FR-α and FR-β staining is shown. (**B**) Summary of the percentage of tissue sections staining positive for FR-α and FR-β (*n* = 64).

In order to explore the expression patterns of the FR isoforms in an unrelated tumor type, pancreatic cancer sections were similarly stained and analyzed. As shown in Figure 4, 95% of all tissue sections stained positive for either FR-α or FR-β. However, in contrast to the staining pattern seen in lung cancer, roughly half of the tumor sections stained positive for only one FR isoform (i.e. with most of this subset staining positive solely for FR-β). Although very few pancreatic cancers stained positive for only FR-α, roughly 45% expressed substantial numbers of both FR-α and FR-β.

**Figure 5:**
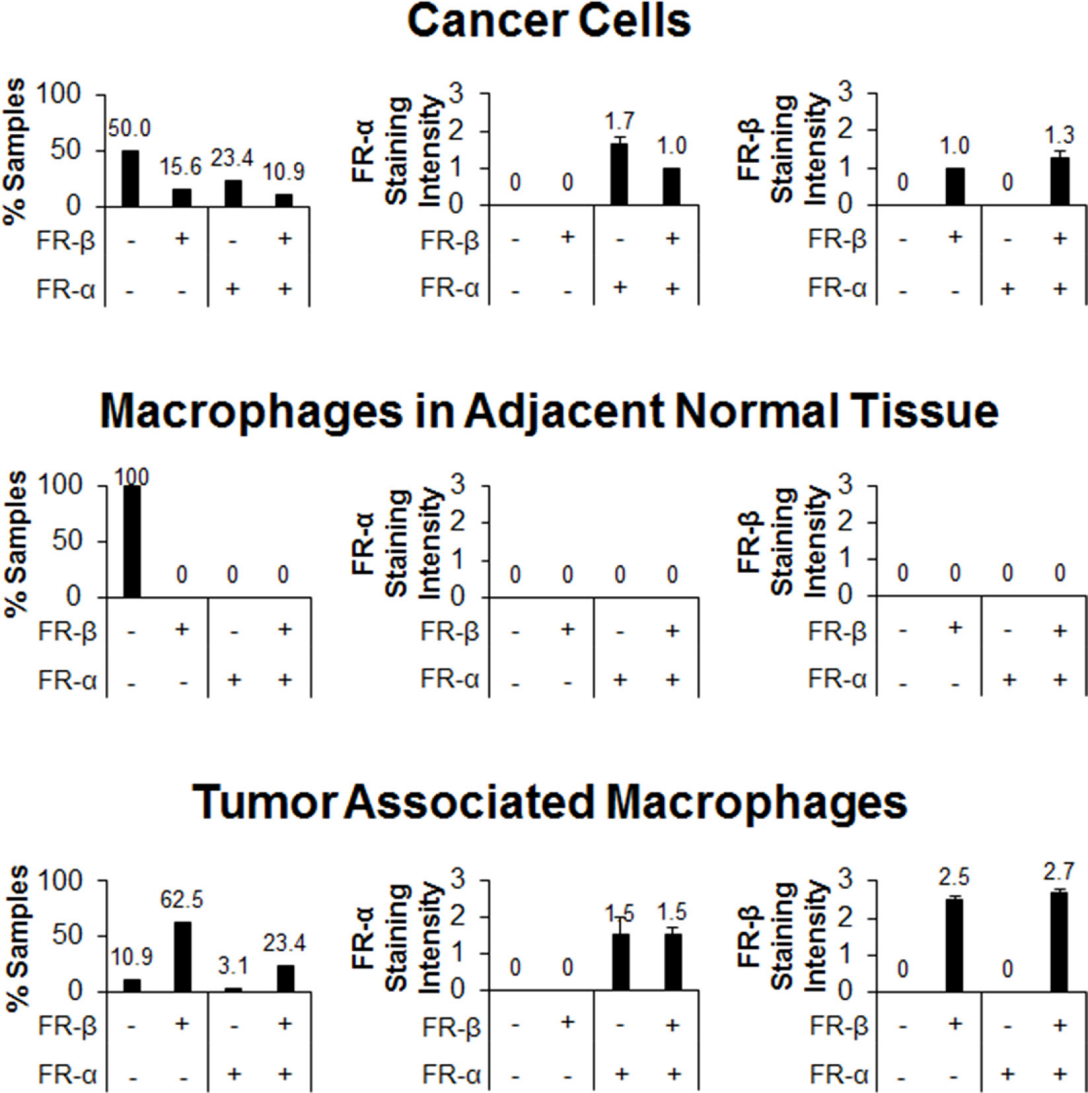
FR-α and FR-β expression in different cell populations within pancreatic cancer tissue samples Sequential sections were stained with mAb343 and m909. Staining of macrophages, normal cells, and cancer cells were graded on the same 0 to 3 scale (*n* = 64). The first column is a summary of the percentage of tissue sections that stained positive in each FR staining group (FR-α low/FR-β low, FR-α low/FR-β high, FR-α high/FR-β low, and FR-α high/FR-β high). The second and third columns show the average staining intensity of FR-α and FR-β, respectively, in each of the four staining groups (error bars represent SEM).

To more precisely characterize the specific cells that carry the FR-α and FR-β antigens, each FR^+^ and FR^−^ staining cell was carefully scrutinized for its specific cell type and location, and then categorized as either a cancer cell, macrophage-like cell located within the tumor margin, macrophage-like cell located outside the tumor margin, or other normal cell (endothelial cell, fibroblast, etc.). When only normal cells outside the tumor margins were analyzed, FR-α was present on >80% of the samples examined where it was found almost exclusively on ductal epithelial cells. Very weak FR-β staining was also observed outside of the tumor boundaries in >60% of sections, with most of this staining found on either glandular cells or ductal epithelial cells. Curiously, macrophages found in these normal tissue regions were completely devoid of both FR-α and FR-β staining (Figure 5), suggesting they were neither activated to a pro- or anti-inflammatory phenotype.

More focused analyses of cancer cells in each of the tumor sections demonstrated that ~50% of sections showed no FR-α or FR-β staining. Moreover, the intensities of those cancer cells that did stain positive for either FR-α or FR-β was relatively low, with an average staining intensity of 1.7 and 1.0, respectively (Figure 5). Additionally, staining of cancer cells was heterogeneous even among the same tissue section with an average relative standard deviation of 23% and a range between ~12% and 45% when grayscale values of cells were quantified. When tumor associated macrophages were specifically examined, 26% were found to express FR-α while 86% were positive for FR-β. Not surprisingly, the staining intensity of FR-β was much greater in the tumor associated macrophages than in any other cells examined (an average intensity of ~2.5; Figure 5).

One of the more surprising observations was that staining of neither FR-α nor FR-β was limited to the cell type in which it is normally expressed. That is, although FR-α is normally observed on cancer cells [10–21] and a minor subset of healthy epithelial cells [2–4], FR-α was also observed on a subpopulation of tumor-associated macrophages. Similarly, while FR-β expression was normally thought to be restricted to the activated subset of myeloid cells (i.e. pro- and anti-inflammatory monocytes and macrophages), it was also found on some cancer cells. While aberrant gene expression might be invoked to account for these unanticipated expression patterns, it should also be noted that folate receptors are glycosylphosphatidylinositol-anchored proteins and that GPI-anchored proteins can jump from one membrane to another [38]. Moreover, cancer cells are similarly known to release large quantities of exosomes and/or microparticles that contain cancer cell proteins [39], and these exosomes/microparticles can fuse with adjacent normal cells and thereby transfer their proteins to the normal cell plasma membranes. Obviously, further investigation of this unexpected finding will be required before an unequivocal mechanism of intercellular FR transfer can be determined. However, the data do suggest that some sharing of membrane components likely occurs among cells within a tumor mass and that this sharing can alter the antigen staining pattern with the tumor.

## DISCUSSION

To the best of our knowledge, this is the first delineation of the independent expression of FR-α and FR-β in the same tumor mass. Our analysis shows that all four combinations of high and low FR-α and FR-β expression can be found in any single cancer type and that expression can be heterogeneous, even within the same tumor sample. Taken together, this suggests that the intensity of an FR-targeted imaging agent cannot be accurately exploited to indicate the level of FR-α expression on a tumor's cancer cells. This conclusion is especially important when the imaging agent is used to select patients for treatment with a folate receptor-targeted therapy, since delivery of a folate-linked therapeutic agent to a tumor whose imaging agent uptake is dominated by FR-β expressing macrophages may have little therapeutic impact on the patient's tumor. Additionally, the heterogeneity within the tumor could result in a strong selective pressure for the survival and proliferation of non or low FR-expressing malignant cells, possibly resulting in recurrence of disease.

In contrast to the above scenario, when a folate-linked imaging agent is exploited for tumor localization, staging, intra-operative imaging, or analysis of cancer recurrence, the added signal intensity arising from imaging agent uptake by both tumor cells and tumor-associated macrophages can augment the sensitivity of the procedure. For example, >65% of pancreatic tumors exhibited no FR-α cancer cell staining, but >85% of samples had FR-β positive tumor associated macrophages and ~25% had FR-β positive cancer cells. An imaging agent that would solely image FR-α might be totally inadequate for pancreatic cancer detection, but an FR isoform agnostic imaging agent might have the sensitivity to detect most if not all malignant lesions. Similarly, although most nonsmall cell lung cancers strongly express FR-α, a subset expresses no FR-α, but does express FR-β. In the case of fluorescence guided surgeries in which localization and resection of malignant lesions has been guided by an FR-targeted fluorescent dye, a number of tumor nodules have indeed been discovered that have expressed FR-β but not FR-α [40]. Because failure to remove these hidden malignant lesions can lead to disease progression and death, the ability to exploit the added sensitivity contributed by FR-β imaging could conceivably be life-saving.

## MATERIALS AND METHODS

### Materials

Anti-mouse IgG-PE antibody conjugates and anti-human IgG-PE antibody conjugates were purchased from BioLegend (San Diego, CA). Anti-mouse, anti-human, and anti-rabbit HRP-conjugated secondary antibodies were obtained from Jackson ImmunoResearch Laboratories (West Grove, PA). HRP luminescent substrate was acquired from Biorad (Hercules, CA). Horseradish peroxidase (HRP)-streptavidin and EZ-Link Sulfo-NHS-LC-Biotin were purchased from Thermo Scientific (Madison, WI). Ethanol, xylene, hydrogen peroxide and Tween-20 procured from Sigma-Aldrich (St. Louis, MO). Target Retrieval buffer was obtained from DAKO (Carpinteria, CA). The cancer tissue microarrays examined consisted of a pancreatic cancer microarray from the Translational Genomics Research Institute (Phoenix, AZ), a custom multi-tumor tissue microarray (TMA-00300) from Asterand (Detroit, MI) and a lung cancer microarray from University of Pennsylvania (Philadelphia, PA). Cell culture reagents and all other materials were purchased from VWR (Chicago, IL).

### Antibody

A previously described murine monoclonal antibody raised against human FR-α (mAb343) was used for IHC assessment of FR-α expression [41]. To query FR-β expression, a human monoclonal anti-human FR-β antibody (m909) was generated against the extracellular domain of human FR-β and has been characterized previously in human samples [42]. Antibodies mAb343 and m909 were labeled with EZ-Link Sulfo-NHS-LC-Biotin according to manufacturer's instructions. The specificity of biotinylated-m909 for human FR-β using this method has been established previously [22].

### Cell culture

MDA-MB-231 and CHO-β (CHO cells transfected with a FR-β expression vector) were cultured in RPMI 1640 media supplemented with 10% fetal bovine serum and 1% penicillin-streptomycin at 37°C in a humidified 95% air – 5% CO_2_ atmosphere. Cells were split into a ~1:8 ratio when flasks reached confluence. All testing was performed by the 10th passage.

### Flow cytometry

MDA-MB-231 and CHO-β cells were dislodged from culture flasks by mechanical scraping in PBS, and cell density was adjusted to 1 × 10^6^ cells / mL. 0.1 mL of cells were incubated in the presence or absence of 0.1 μg/mL mAb343 or m909 for 30 min at room temperature. mAb343 treated cells were washed in PBS and then incubated in anti-mouse IgG-PE conjugate for 30 min at room temperature and m909 treated cells were similarly washed and incubated in anti-human IgG-PE conjugate. Cells were washed in PBS followed by incubation with 7-AAD for 15 min at room temperature. Cells were then analyzed via flow cytometry (BD Biosciences Accuri C6; San Jose, CA). Live cells were gated and the amount of secondary antibody fluorescence was recorded.

### Western blotting

MDA-MB-231 and CHO-β cells were dislodged from culture flasks by mechanical scraping in PBS and cell density was adjusted to 1 × 10^6^ cells / mL. Cells were lysed in loading buffer and 20 μL was added to each well of a 10% polyacrylamide gel. After electrophoresis, proteins were transferred to nitrocellulose paper and the membrane was blocked with 5% BSA overnight at 4°C. After washing with tris-buffered saline + 0.05% Tween 20 (TBST), membranes were incubated with mAb343 (1:10,000), m909 (1:5000), or anti-actin (1:20,000) in TTBS overnight at 4°C. The labeled membrane was washed in TBST and then incubated with anti-mouse (1:20,000), anti-human (1:5000), or anti-rabbit (1:5000) HRP-conjugated secondary antibody for 1 hour at room temperature for mAb343, m909, and anti-actin treated blots, respectively. Blots were washed with TBST and developed in luminescent HRP substrate prior to imaging using a ProteinSimple FluorChem R camera system (San Jose, CA).

### Immunohistochemistry

All tumor microarrays (TMA) were supplied by the provider fixed in formalin and embedded in paraffin. TMA samples were deparaffinized with xylene (3 changes), rehydrated in a series of ethanol dilutions (100%, 95%, then 70%) and rinsed in distilled water. TMA slides then were placed in a preheated DAKO Target Retrieval buffer for 40 min, cooled in the same buffer for 20 min and rinsed for 5 min in distilled water. After the heat inactivated epitope retrieval step, TMA sections were incubated in 3% H_2_O_2_ in ethanol for 5 min to inactivate the endogenous peroxides, followed by a protein blocking step for 5 min. Slides were rinsed with Tris-buffered saline containing 0.05% Tween 20 (TBST) wash buffer and incubated for 30 min at room temperature with biotinylated-m909 or biotinylated-mAb343 (0.07 mg/mL). TMA sections then were rinsed with TBST wash buffer and incubated with HRP-streptavidin for 10 min at room temperature. The slides then were washed with TBST and incubated in 3,3′-diaminobenzidine for 5 min at room temperature, counterstained with Modified Schmidts's Hematoxylin for 5 min and rinsed in tap water for 3 min. The samples then were dehydrated through graded alcohols, cleared in 3 changes of xylene and mounted with a permanent mounting media. Tumor sections were analyzed by trained pathologists and the staining intensity was scored on a scale of 0 to 3.

### Statistics

Differences between groups were determined by either a *t*-test (assuming equal variance and 2-tails for all samples) for data sets with only 2 groups or a 1-way ANOVA for data sets containing multiple groups. Correlation analyses were performed using a Spearman correlation analysis. Differences were considered statistically significant if the *p*-value was < 0.05.

## Abbreviations

FR: folate receptor
TMA: tumor microarray
